# Complete Genome Sequencing of Dengue Virus Type I from Zhuhai City, China

**DOI:** 10.1128/genomeA.01686-15

**Published:** 2016-02-11

**Authors:** Chao Chen, Quande Wei

**Affiliations:** a Engineering School, Beijing Normal University, Zhuhai, China; b Zhuhai Centre for Disease Control and Prevention, Zhuhai, China

## Abstract

The detection and successful typing of dengue virus (DENV) from patients with suspected dengue fever are important for stopping outbreaks and preventing the recurrence of this virus. In this study, we reported complete genomic sequences of DENV-1 isolated from Zhuhai patients, providing basic information for future epidemic dengue disease detection.

## GENOME ANNOUNCEMENT

Dengue viruses (DENVs) are mosquito-borne viruses that have emerged since World War II and have now become the most important arthropod-borne viral infection in humans (1, 2). The diseases it causes include dengue fever (DF), dengue hemorrhagic fever (DHF), and dengue shock syndrome (DSS). DENVs have five antigenically distinct serotypes (DENV-1 to -5), which are distributed in tropical and subtropical areas (3, 4). In China, outbreaks of DF have occurred mainly in coastal provinces since 1978, of which Guangdong Province is the major affected area. Most outbreaks of DF in Guangdong Province were caused by DENV-1 from a 1995 outbreak. Here, we determined the complete genome sequences of DENV-1 that caused the DF outbreak at Zhuhai, China, in 2007.

Acute blood samples from 41 patients were collected between days 2 and 7 after the onset of dengue fever syndrome in the summer of 2007 at Zhuhai city. Viral RNA was extracted from the serum samples using the QIAamp viral RNA minikit (Qiagen, Hilden, Germany), according to the manufacturer’s instructions. The primers used for amplification and sequencing of the DENV-1 genomic sequences were designed based on the GenBank accession no. AB178040. PCR products were checked on a 1% agarose gel and then purified for sequencing in an ABI 3730xl genetic analyzer (ABI, St. Louis, MO).

One representative of a related DENV-1 positive-strand RNA virus was subjected for whole-genome sequencing. The virus ZH1067/07 has an RNA genome of 10,735 nucleotides. It consisted of 94 nucleotides at the 3′ noncoding region, 462 nucleotides at the 5′ noncoding region, and an open reading frame (ORF) located from nucleotides 95 to 10273. The most closely related strains to ZH1067/07 were AB178040 (99% similarity, isolated in Japan and Micronesia in 2004), Fj231/04 (99% similarity, isolated in Fujian, China, in 2004), and AY726554 (98% similarity, isolated in Myanmar in 2004). Epidemiological analysis showed that the first DENV-1 patient in a dengue outbreak in Zhuhai 2007 developed DEN symptoms after his return to Zhuhai from Fujian, suggesting the DEN outbreak in Zhuhai may be caused by an imported ZH1067/07-like virus from Fujian or even Japan or Myanmar. However, more detailed clues or molecular epidemiology study is needed to understand the epidemiology of DENV-1, which reemerged from 2011 to 2014 at Zhuhai.

### Nucleotide sequence accession numbers.

This whole-genome shotgun project has been deposited at DDBJ/EMBL/GenBank under the accession no. EU359008. The version described in this paper is the first version, EU359008.1.
